# Unveiling gender disparities in mental health among Chinese preschoolers: partial statistical mediation by parental overreactivity

**DOI:** 10.3389/fpsyt.2026.1876731

**Published:** 2026-08-19

**Authors:** Ziyi Wang, Jiahua Liu, Hongli Sun, Xiangdi Yang, Jie Mi

**Affiliations:** 1 School of Humanities and Social Science, Xi'an Jiaotong University, Xi’an, Shaanxi, China; 2 Department of Clinical Laboratory, Xi’an Children’s Hospital, Affiliated Children’s Hospital of Xi’an Jiaotong University, National Regional Children’s Medical Center (Northwest), Xi’an, Shaanxi, China; 3 Shaanxi Institute for Pediatric Diseases, Xi’an Key Laboratory of Children’s Health and Diseases, Xi’an Children’s Hospital, Affiliated Children’s Hospital of Xi’an Jiaotong University, National Regional Children’s Medical Center (Northwest), Xi’an, Shaanxi, China; 4 Department of Stomatology, Xi’an Children’s Hospital, Affiliated Children’s Hospital of Xi’an Jiaotong University, National Regional Children’s Medical Center (Northwest), Xi’an, Shaanxi, China; 5 Department of Pharmacy, Xi’an Children’s Hospital, Affiliated Children’s Hospital of Xi’an Jiaotong University, National Regional Children’s Medical Center (Northwest), Xi’an, Shaanxi, China

**Keywords:** child mental health, gender disparities, mediation analysis, parental overreactivity, strengths and difficulties questionnaire

## Abstract

**Objectives:**

This study aimed to examine whether parental overreactivity is associated with the relationship between child gender and mental health in preschool children, exploring potential pathways that may underlie observed gender-related disparities.

**Methods:**

A total of 21,366 children aged 3–6 years were recruited through stratified cluster sampling from 189 kindergartens across 13 districts in western China. Child gender (boy/girl) was treated as the exposure variable. Parental overreactivity was assessed using the Arnold–O’Leary Parenting Scale, and child mental health was measured with the Strengths and Difficulties Questionnaire (SDQ), focusing on total difficulties and prosocial behavior. Multivariable logistic and linear regression models were applied, and mediation analysis was conducted to quantify indirect effects.

**Results:**

Compared with boys, girls exhibited a 12% lower risk of total difficulties (OR = 0.88; 95% CI: 0.82–0.94; P = 0.0003) and a 21% higher likelihood of prosocial behavior (OR = 1.21; 95% CI: 1.14–1.28; P < 0.0001). Each one-point increase in parental overreactivity was associated with an 85% higher risk of total difficulties (OR = 1.85; 95% CI: 1.78–1.92; P < 0.0001) and a 34% reduction in prosocial behavior (OR = 0.66; 95% CI: 0.64–0.68; P < 0.0001). Girls experienced significantly lower levels of parental overreactivity (β = –0.04; 95% CI: –0.06 to –0.01; P = 0.0152). Mediation analysis showed that overreactivity statistically explained 16.22% of the gender effect on total difficulties and 7.49% on prosocial behavior. Subgroup analyses suggested stronger effects among families with lower socioeconomic status.

**Conclusions:**

Parental overreactivity was identified as a statistically significant indirect factor in the association between child gender and early mental health. Girls showed lower parental overreactivity, which was statistically associated with more favorable psychological profiles. These findings suggest that parental overreactivity may represent a potential intervention target, and highlight the value of gender-sensitive parenting approaches for narrowing early mental health disparities.

## 1 Introduction

Child mental health denotes a developmental state in which children are able to achieve age-appropriate emotional, social, and cognitive growth. The World Health Organization emphasizes that mental health is not merely the absence of psychiatric disorders, but a condition that allows individuals to realize their abilities, manage stress, function productively, and contribute to society (1). Optimal child mental health encompasses both the absence of psychological difficulties and the presence of positive competencies such as emotional regulation, social skills, and prosocial behaviors (2). Both these competencies and mental health challenges significantly influence learning, behavior, and overall health outcomes throughout childhood, with lasting effects into adolescence and adulthood (1). Notably, mental health problems emerging in early childhood are associated with an increased risk of lifelong mental disorders, posing substantial burdens on the child, their family, and society at large (3, 4). Consequently, early detection and targeted intervention during preschool years are crucial.

Gender, as a system of sociocultural, psychological and behavioral attributes linked to biological sex (5), plays a critical role in shaping mental health outcomes. Research consistently demonstrates significant gender disparities in the occurrence and symptomatic presentation of mental health disorders among children, including behavioral issues, anxiety, attention-deficit/hyperactivity disorder and eating disorders (6, 7). Internalizing problems like depression and anxiety occur more frequently in girls (8, 9), whereas externalizing behaviors, including aggression and rule-breaking, are more common among boys (5, 10). These early-onset mental health issues contribute substantially to the global disease burden (11), entail long-term economic costs (12), and frequently persist into adulthood (3, 13). Despite these well-documented disparities, the factors driving these disparities remain insufficiently explained.

Overreactive parenting refers to controlling, aversive, and intrusive responses to children’s problematic behaviors (14), typically characterized by excessive emotional reactions, harsh discipline, or punitive measures. Empirical evidence links parental overreaction to behavioral issues in children, particularly deficits in emotional regulation and social competence and resilience (15–18). Despite these well-established associations, little is known about whether overreactivity serves as a factor that mediates the relationship between child gender and mental health outcomes.

Among various parenting dimensions, we focused on overreactivity for three reasons. First, overreactivity is conceptually distinct from general parenting styles (e.g., authoritarian or authoritative parenting) in that it captures context-specific dysregulated responses to child misbehavior rather than global parenting attitudes (19), making it more amenable to behavioral intervention. Second, empirical evidence has linked overreactive parenting to children’s internalizing and externalizing behavioral problems during early childhood (16), and longitudinal studies have identified overreactive discipline as a predictor of young adult adjustment (17). Third, gender-differentiated patterns in parental discipline have been documented: meta-analytic evidence suggests that physical punishment is applied more frequently to boys than girls in some cultural contexts (20), and parents have been found to use more supportive speech with daughters than with sons (21). These considerations led us to hypothesize that overreactivity might statistically account for part of the gender–mental health association.

The present study had both confirmatory and exploratory aims. For the confirmatory aims, we tested three hypotheses based on prior literature: (1) girls would show lower levels of total difficulties and higher levels of prosocial behavior than boys; (2) higher parental overreactivity would be associated with higher total difficulties and lower prosocial behavior; and (3) girls would experience lower levels of parental overreactivity than boys. For the exploratory aim, we examined whether parental overreactivity could statistically mediate the associations between child gender and the two mental health outcomes, given that prior studies had not directly tested this mediation pathway. Using a large cross-sectional sample, we sought to provide descriptive evidence regarding how parental discipline styles might statistically relate to gender differences in early mental health. If these associations are replicated in future longitudinal studies, they may suggest that reducing parental overreactivity could be a potential focus for family support programs.

## 2 Methods

### 2.1 Study population

This investigation was carried out in collaboration with the municipal Education Bureau and participating kindergartens in a western region of China. A stratified cluster sampling strategy was adopted. The city was divided into 13 administrative districts and counties, from which public kindergartens were randomly selected using the official registry. The number of schools and participants from each district was proportionally determined according to the child population. A total of 189 kindergartens were recruited, and all preschoolers aged 3–6 years and their parents were invited to take part. Data collection occurred between February 28 and March 5, 2025. Informed consent was provided from one parent or guardian per child before participation. Among 28,000 questionnaires distributed, 25,017 were returned, yielding a response rate of 89.3% (25,017/28,000). Exclusions were applied for missing or implausible parental age (517), non-parent respondents (2,408), and missing or inconsistent child age (726), leaving 21,366 valid cases for analysis. In this final analytical sample, all variables included in the regression models had no missing values. The intraclass correlation coefficients (ICCs) for all outcomes were negligible (ICC < 0.001 for total difficulties, and ICC = 0 for both prosocial behavior and parental overreactivity; see **Supplementary Table S1**), indicating that kindergarten-level clustering did not meaningfully contribute to the total variance. Given that the ICC values were essentially zero, standard regression models (rather than multilevel models) were used for all primary analyses, as the negligible cluster-level variance indicates that kindergarten clustering does not meaningfully affect parameter estimates or standard errors. To assess the robustness of our findings to missing data, we performed multiple imputation using the MICE algorithm with 10 imputations (20 iterations each) on the pre-exclusion sample of 25,017 returned questionnaires. The results were virtually identical to those obtained from the complete-case analysis (**Supplementary Table S2**), indicating that missing data did not bias our conclusions.

### 2.2 Exposure and mediator

The main exposure variable was child gender (boy/girl), obtained from parent-completed questionnaires. The mediating factor under investigation was parental overreactivity, measured using the Arnold-O’Leary Parenting Scale. The Parenting Scale’s overreactivity subscale (19) was utilized to measure dysfunctional parenting behaviors characterized by excessive emotional responses, focusing on parents’ tendencies to exhibit anger, frustration, or harsh reactions to children’s behavioral issues (17). It includes 10 items rated on a 7-point Likert scale (1 = effective strategies, 7 = highly punitive responses), with higher scores reflecting greater overreactivity (16). For example, one item asks parents to rate their response when their child misbehaves, anchored by 1 = “I speak to my child calmly” and 7 = “I raise my voice or yell.” In this study, we used the item-average score across the 10 items (range 1–7) rather than the sum score. The scale shows robust internal consistency and has been validated for use with parents of preschoolers and young adolescents (19, 22). It is also suitable for evaluating parenting strategies in families with children facing developmental or behavioral difficulties (16, 23). In our study, the Parenting Scale demonstrated satisfactory internal consistency (Cronbach’s α = 0.962). Although originally developed in Western settings (19), the Parenting Scale has shown acceptable psychometric properties in Asian countries, including China (24, 25).

### 2.3 Outcome

Child mental health was measured using the Strengths and Difficulties Questionnaire (SDQ), a validated 25-item instrument. Responses were rated on a three-point scale (0 = “not true” to 2 = “certainly true”). The SDQ yields five subscales: emotional symptoms, conduct issues, hyperactivity, peer challenges, and prosocial behavior. Using established SDQ cutoff values for China (26, 27), the total difficulties score is derived from four domains: emotional symptoms, conduct issues, hyperactivity, and peer challenges. A score above 14 indicates a potential mental health concern, while a prosocial behavior score below 6 suggests limited prosocial tendencies. The SDQ demonstrates strong internal consistency and validity for assessing mental health in Chinese children aged 3 to 17 (27, 28). In our study, the SDQ demonstrated satisfactory internal consistency (Cronbach’s α = 0.703 for the total difficulties scale and 0.826 for the prosocial behavior scale).

### 2.4 Covariates

To account for potential confounding factors, the following variables were collected: parent gender (male/female), parent age (continuous or categorized as < 40, ≥ 40 years), child age (continuous or grouped as < 4, ≥ 4 to < 5, ≥ 5 years), parent education level (≤ high school, junior college, ≥ bachelor’s degree), employment status (employed/unemployed), yearly household income (< ¥100,000, ≥ ¥100,000), smoking status (yes/no), alcohol consumption (yes/no), and marital status (married/cohabiting, other).

### 2.5 Statistical analysis

Baseline characteristics were grouped by child gender (boys/girls). Group differences were examined using the Kruskal–Wallis H test for continuous variables and Fisher’s exact test for categorical variables. The Kruskal-Wallis test was used because the continuous variables did not meet the normality assumption for parametric tests. It yields identical results to the Mann-Whitney U test for two-group comparisons. Logistic regression models estimated the association between child gender and child mental health, with results presented as odds ratios (OR) and 95% confidence intervals (CI). Associations between parental overreactivity and child mental health were assessed similarly. Linear regression was applied to evaluate differences in mean overreactivity scores by child gender, with results presented as β coefficients and 95% CI. Regression models were constructed: the unadjusted model excluded covariates, while the adjusted model included all sociodemographic variables. Multicollinearity among the independent variables was assessed using variance inflation factors (VIF). All VIF values were below 2.5 (see **Supplementary Table S3**), indicating no serious multicollinearity. Subgroup analyses were performed across parental and household characteristics. To assess whether the findings were sensitive to the choice of dichotomized versus continuous outcomes, and to provide a more nuanced characterization of gender differences across SDQ subdomains, we repeated all regression analyses using continuous SDQ total difficulties and subscale scores (emotional symptoms, conduct issues, hyperactivity, peer challenges, and prosocial behavior) as outcomes. These sensitivity analyses are presented in **Supplementary Table S4**.

Mediation analysis was conducted to test whether parental overreactivity was statistically associated with part of the association between child gender and child mental health. Child gender was entered as the exposure variable, overreactivity as the continuous mediator, and children’s total difficulties and prosocial behavior score as outcome variables. The analysis calculated total, direct, and indirect effects, with the mediation ratio determined using the formula: mediation ratio = (indirect effect/total effect) × 100% to quantify the mediator’s influence (29). Because the data are cross-sectional, the mediation analysis does not establish temporal precedence or causal direction. The indirect effects reported are therefore interpreted as statistical associations that are consistent with a mediation hypothesis, not as causal effects. We emphasize that the indirect effects reported are intended to quantify the degree of statistical association consistent with mediation and to generate hypotheses for future longitudinal studies.

All analyses were conducted using R software (http://www.r-project.org, The R Foundation) and EmpowerStats (http://www.empowerstats.com, X&Y Solutions, Inc., Boston, MA). A two-tailed P < 0.05 was considered statistically significant.

## 3 Results

### 3.1 Baseline characteristics

The study included 21,366 preschool children, comprising 11,062 boys (51.79%) and 10,304 girls (48.21%). Baseline characteristics stratified by child gender are shown in **Table 1**. Significant differences were presented between boys and girls in child age (4.83 ± 0.89 vs. 4.80 ± 0.88 years, P = 0.015), parental age (34.82 ± 4.54 vs. 34.67 ± 4.58 years, P = 0.016), parental gender (24.60% male vs. 23.17% male, P = 0.014), education level (P = 0.009), and overreactivity score (2.92 ± 1.08 vs. 2.88 ± 1.08, P = 0.009). Notably, boys exhibited higher rates of total difficulties (>14: 19.57% vs. 17.60%, P < 0.001) and lower prosocial behavior scores (<6: 51.64% vs. 47.03%, P < 0.001) compared to girls. No significant gender differences were observed for parental employment (P = 0.700), annual family income (P = 0.608), smoking (P = 0.247), marital status (P = 0.132), or alcohol intake status (P = 0.240).

**Table 1:** General baseline characteristics of participants.

Characteristics	All	Boys	Girls	P-value
	N=21366	N = 11062	N = 10304	
Child age (years)	4.82 ± 0.89	4.83 ± 0.89	4.80 ± 0.88	0.015
Parental age (years)	34.75 ± 4.56	34.82 ± 4.54	34.67 ± 4.58	0.016
Parental gender				0.014
Male	5108 (23.91%)	2721 (24.60%)	2387 (23.17%)	
Female	16258 (76.09%)	8341 (75.40%)	7917 (76.83%)	
Education level				0.009
≤ High school diploma	9834 (46.03%)	5093 (46.04%)	4741 (46.01%)	
junior college	5383 (25.19%)	2868 (25.93%)	2515 (24.41%)	
≥ Undergraduate degree	6149 (28.78%)	3101 (28.03%)	3048 (29.58%)	
Employment status				0.700
Working	15642 (73.21%)	8086 (73.10%)	7556 (73.33%)	
Not working	5724 (26.79%)	2976 (26.90%)	2748 (26.67%)	
Annual family income, in thousands, ¥				0.608
< 100	17238 (80.68%)	8910 (80.55%)	8328 (80.82%)	
≥ 100	4128 (19.32%)	2152 (19.45%)	1976 (19.18%)	
Smoking status				0.247
No	18187 (85.12%)	9386 (84.85%)	8801 (85.41%)	
Yes	3179 (14.88%)	1676 (15.15%)	1503 (14.59%)	
Marital status				0.132
Married/Living with partner	20761 (97.17%)	10767 (97.33%)	9994 (96.99%)	
Others	605 (2.83%)	295 (2.67%)	310 (3.01%)	
Alcohol intake status				0.240
No	17858 (83.58%)	9214 (83.29%)	8644 (83.89%)	
Yes	3508 (16.42%)	1848 (16.71%)	1660 (16.11%)	
Overreactivity score	2.90 ± 1.08	2.92 ± 1.08	2.88 ± 1.08	0.009
Total difficulties score				<0.001
≤ 14	17388 (81.38%)	8897 (80.43%)	8491 (82.40%)	
> 14	3978 (18.62%)	2165 (19.57%)	1813 (17.60%)	
Prosocial behavior score				<0.001
< 6	10558 (49.41%)	5712 (51.64%)	4846 (47.03%)	
≥ 6	10808 (50.59%)	5350 (48.36%)	5458 (52.97%)	

Continuous variables are expressed as means ± standard deviations and categorical data using number (percentage), *P < 0.05.

### 3.2 Associations between child gender and mental health and overreactivity

After controlling for parental and sociodemographic covariates, girls had 12% lower risk of total difficulties (OR = 0.88; 95% CI 0.82–0.94; P = 0.0003) and 21% greater likelihood of prosocial behavior (OR = 1.21; 95% CI 1.14–1.28; P < 0.0001) compared with boys (**Table 2**). Each one-unit increment in parental overreactivity was linked to an 85% higher risk of total difficulties (OR = 1.85; 95% CI 1.78–1.92; P < 0.0001) and a 34% reduction in higher prosocial behavior (OR = 0.66; 95% CI 0.64–0.68; P < 0.0001). In linear regression, girls scored on average 0.04 points lower on the overreactivity scale than boys (β = –0.04; 95% CI –0.06 to –0.01; P = 0.0152) after adjustment (**Table 3**). Given the large sample size, this small effect size should be interpreted with caution, as statistical significance does not necessarily imply clinical relevance. It should also be noted that the overreactivity score was calculated as the item-average across 10 items (range 1–7), so the β value represents the mean difference per item on a 7-point scale. Sensitivity analyses using continuous SDQ scores were consistent with the primary categorical findings (**Supplementary Table S4**). Girls scored significantly lower than boys on total difficulties, conduct issues, hyperactivity, and peer challenges, but slightly higher on emotional symptoms, reflecting the well-documented pattern of higher internalizing problems in girls (8, 9). The largest gender differences were observed for hyperactivity and peer problems. Prosocial behavior remained significantly higher in girls.

**Table 2:** Associations between child gender and overreactivity score and mental health in preschool children.

Exposure	Non-adjusted model	Adjust model
Total difficulties		
Child gender		
Boys	Ref.	Ref.
Girls	0.88 (0.82, 0.94) 0.0002	0.88 (0.82, 0.94) 0.0003
Overreactivity score	1.85 (1.78, 1.93) <0.0001	1.85 (1.78, 1.92) <0.0001
Prosocial behavior		
Child gender		
Boys	Ref.	Ref.
Girls	1.20 (1.14, 1.27) <0.0001	1.21 (1.14, 1.28) <0.0001
Overreactivity score	0.67 (0.65, 0.68) <0.0001	0.66 (0.64, 0.68) <0.0001

The data is represented by OR (95% CI) P value.

Non-adjusted model adjust for: None.

Adjust model adjust for: parental gender; parental age; child age; smoking status; alcohol intake status; education level; annual family income; employment status; marital status.

**Table 3:** Associations between child gender and overreactivity score in preschool children.

Exposure	Non-adjusted model	Adjust model
Child gender		
Boys	Ref.	Ref.
Girls	-0.04 (-0.07, -0.01) 0.0088	-0.04 (-0.06, -0.01) 0.0152

The data is represented by β (95% CI) P value.

Non-adjusted model adjust for: None.

Adjust model adjust for: parental gender; parental age; child age; smoking status; alcohol intake status; education level; annual family income; employment status; marital status.

### 3.3 Stratified analyses and interactions test

**Figure 1:**
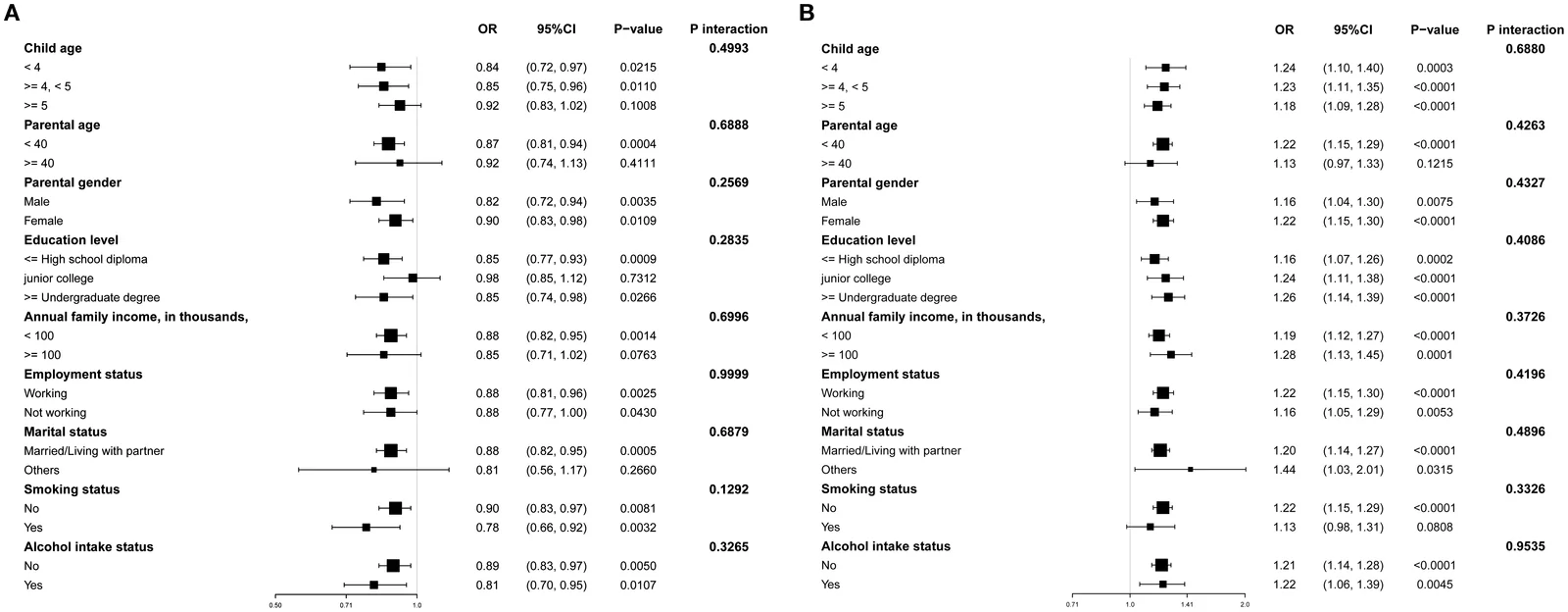
**(A)** Stratified analysis between child gender and the risk of total difficulties in children. Adjusted for all covariates except the stratification variable itself (see full covariate list in Methods, section 2.4). **(B)** Stratified analysis between child gender and higher prosocial behavior in children. Adjusted for all covariates except the stratification variable itself (see full covariate list in Methods, section 2.4).

Stratified models consistently demonstrated protective associations for girls across most subgroups (**Table 4**). **Figure 1** illustrated gender differences in child outcomes across strata defined by parental and household characteristics. For total difficulties(**Figure 1A**), the protective effect of female children was particularly pronounced among children aged <5 years (< 4 years: OR = 0.84, 95% CI: 0.72–0.97, P = 0.0215; ≥ 4 to < 5 years: OR = 0.85, 95% CI: 0.75–0.96, P = 0.0110), but was not statistically significant among children ≥5 years (OR = 0.92, 95% CI: 0.83–1.02, P = 0.1008). For parental age <40, girls had 13% lower risk of total difficulties (OR = 0.87, 95% CI: 0.81–0.94, P = 0.0004) compared with boys. However, the association was not significant in parental age ≥ 40 (OR = 0.92, 95% CI: 0.74–1.13, P = 0.4111). The association was significant for parents with high school education or below (OR = 0.85, 95% CI: 0.77–0.93, P = 0.0009) or undergraduate degree or above (OR = 0.85, 95% CI: 0.74–0.98, P = 0.0266). For families with an annual income < ¥100,000, girls had 12% lower risk of total difficulties (OR = 0.88; 95% CI 0.82–0.95; P = 0.0014) compared with boys. The connection was also apparent in the married/living with partner group (OR = 0.88, 95% CI: 0.82–0.95, P = 0.0005) but not in other marital status groups (OR = 0.81; 95% CI 0.56–1.17; P = 0.2660). Similarly, the positive association between female children and prosocial behavior was robust across all strata (P < 0.05 for all subgroups) except for parental age ≥ 40 and parental smoking group (**Figure 1B**). No significant interaction effects were detected across stratification variables (P interaction > 0.05 for all stratification variables).

**Table 4:** Stratified analyses of the association between child gender and mental health in preschool children.

Characteristics	Total difficulties			P interaction	Prosocial behavior			P interaction
	Boys	Girls			Boys	Girls		
		OR (95%CI)	P-value			OR (95%CI)	P-value	
Child age				0.4993				0.6880
< 4	Ref.	0.84 (0.72, 0.97)	0.0215		Ref.	1.24 (1.10, 1.40)	0.0003	
≥ 4 to 5	Ref.	0.85 (0.75, 0.96)	0.0110		Ref.	1.23 (1.11, 1.35)	<0.0001	
≥ 5	Ref.	0.92 (0.83, 1.02)	0.1008		Ref.	1.18 (1.09, 1.28)	<0.0001	
Parental age				0.6888				0.4263
< 40	Ref.	0.87 (0.81, 0.94)	0.0004		Ref.	1.22 (1.15, 1.29)	<0.0001	
≥ 40	Ref.	0.92 (0.74, 1.13)	0.4111		Ref.	1.13 (0.97, 1.33)	0.1215	
Parental gender				0.2569				0.4327
Male	Ref.	0.82 (0.72, 0.94)	0.0035		Ref.	1.16 (1.04, 1.30)	0.0075	
Female	Ref.	0.90 (0.83, 0.98)	0.0109		Ref.	1.22 (1.15, 1.30)	<0.0001	
Education level				0.2835				0.4086
≤ High school diploma	Ref.	0.85 (0.77, 0.93)	0.0009		Ref.	1.16 (1.07, 1.26)	0.0002	
junior college	Ref.	0.98 (0.85, 1.12)	0.7312		Ref.	1.24 (1.11, 1.38)	<0.0001	
≥ Undergraduate degree	Ref.	0.85 (0.74, 0.98)	0.0266		Ref.	1.26 (1.14, 1.39)	<0.0001	
Annual family income, in thousands, ¥				0.6996				0.3726
< 100	Ref.	0.88 (0.82, 0.95)	0.0014		Ref.	1.19 (1.12, 1.27)	<0.0001	
≥ 100	Ref.	0.85 (0.71, 1.02)	0.0763		Ref.	1.28 (1.13, 1.45)	0.0001	
Employment status				0.9999				0.4196
Working	Ref.	0.88 (0.81, 0.96)	0.0025		Ref.	1.22 (1.15, 1.30)	<0.0001	
Not working	Ref.	0.88 (0.77, 1.00)	0.0430		Ref.	1.16 (1.05, 1.29)	0.0053	
Marital status				0.6879				0.4896
Married/Living with partner	Ref.	0.88 (0.82, 0.95)	0.0005		Ref.	1.20 (1.14, 1.27)	<0.0001	
Others	Ref.	0.81 (0.56, 1.17)	0.2660		Ref.	1.44 (1.03, 2.01)	0.0315	
Smoking status				0.1292				0.3326
No	Ref.	0.90 (0.83, 0.97)	0.0081		Ref.	1.22 (1.15, 1.29)	<0.0001	
Yes	Ref.	0.78 (0.66, 0.92)	0.0032		Ref.	1.13 (0.98, 1.31)	0.0808	
Alcohol intake status				0.3265				0.9535
No	Ref.	0.89 (0.83, 0.97)	0.0050		Ref.	1.21 (1.14, 1.28)	<0.0001	
Yes	Ref.	0.81 (0.70, 0.95)	0.0107		Ref.	1.22 (1.06, 1.39)	0.0045	

Adjust for: parental gender; parental age; child age; smoking status; alcohol intake status; education level; annual family income; employment status; marital status. Stratification variables were not involved in the adjustment of the respective stratification analysis.

### 3.4 Mediation analysis

**Figure 2:**
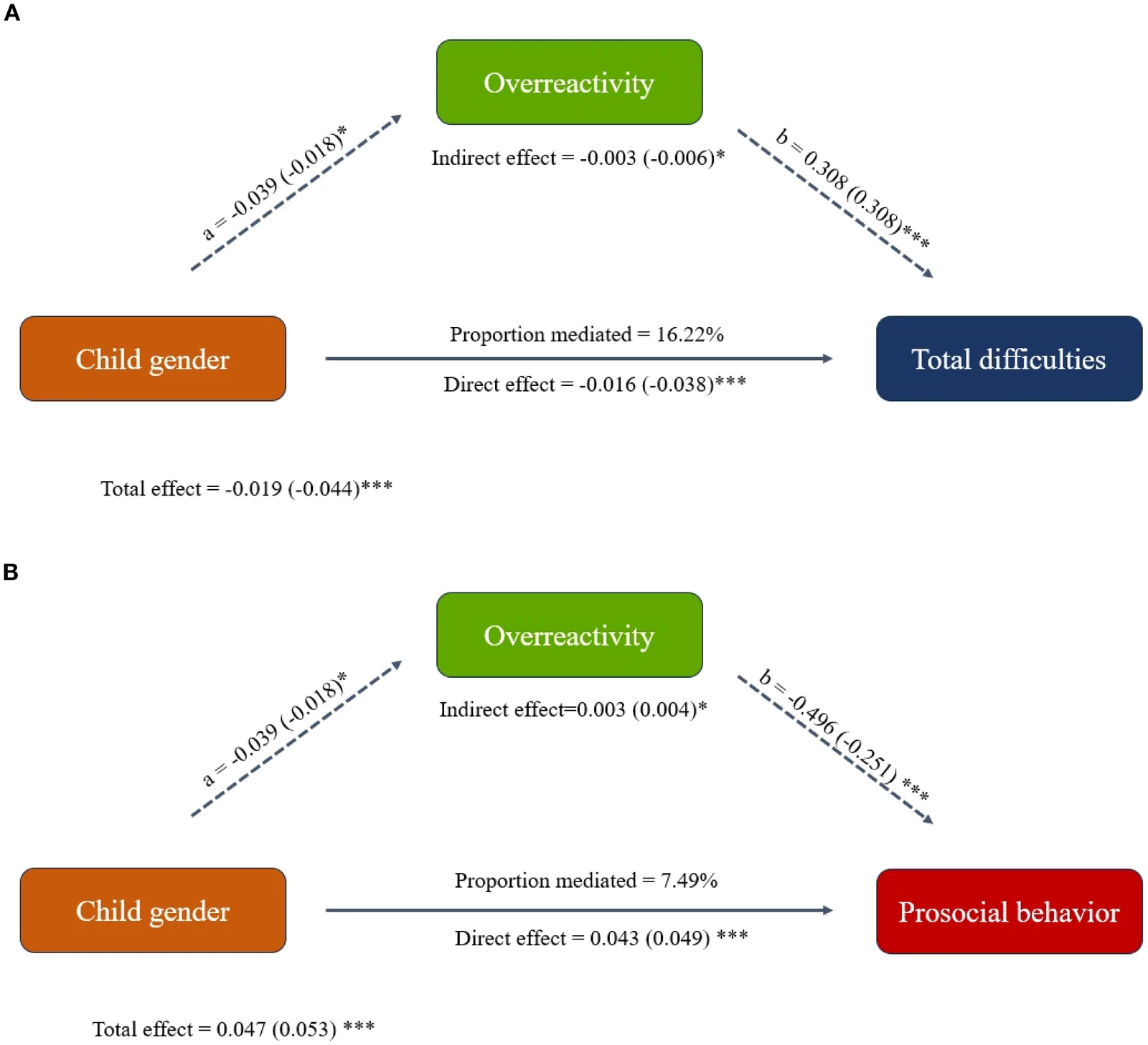
**(A)** Mediation effect of overreactivity in the association between child gender and total difficulties in preschool children. Path coefficients are shown as unstandardized (standardized) estimates. Adjusted for all covariates (see Methods, section 2.4). ***P < 0.001; *P < 0.05. **(B)** Mediation effect of overreactivity in the association between child gender and prosocial behavior in preschool children. Path coefficients are shown as unstandardized (standardized) estimates. Adjusted for all covariates (see Methods, section 2.4). ***P < 0.001; *P < 0.05.

Overreactivity statistically mediated 16.22% of the association between female children and reduced total difficulties (indirect effect: -0.003, 95% CI: -0.005 to -0.001, P = 0.0140), as illustrated in the mediation model pathway of **Figure 2A**. Similarly, it mediated 7.49% of the association with improved prosocial behavior (indirect effect: 0.003, 95% CI: 0.001–0.006, P = 0.0140), shown in **Figure 2B**. The detailed statistical values for these indirect, direct, and total effects were comprehensively presented in **Table 5**. These findings are consistent with a statistical mediation hypothesis, but cross-sectional data preclude causal conclusions.

**Table 5:** Mediation analysis of overreactivity in the association between child gender and mental health in preschool children.

Independent variable	Mediator	Outcome	Total effect	Indirect effect	Direct effect	Proportion mediated, %
Child gender	Overreactivity	Total difficulties	-0.019(-0.029, -0.009) <0.0001	-0.003(-0.005, -0.001) 0.0140	-0.016(-0.025, -0.005) 0.0040	16.22%
		Prosocial behavior	0.047(0.033, 0.060) <0.0001	0.003(0.001, 0.006) 0.0140	0.043(0.030, 0.057) <0.0001	7.49%

Adjust for: parental gender; parental age; child age; smoking status; alcohol intake status; education level; annual family income; employment status; marital status.

## 4 Discussion

The present study provides cross-sectional evidence consistent with the hypothesis that parental overreactivity is statistically associated with gender disparities in preschool mental health. Even after adjusting for a comprehensive set of covariates, girls exhibited a 12% lower risk of total difficulties and a 21% higher prosocial behavior compared to boys, aligning with a substantial body of literature that reported girls generally displayed fewer mental health problems and greater prosocial tendencies than boys (8, 10). Notably, our mediation analysis revealed that parental overreactivity partially accounted for these associations, accounting for 16.22% of the association on total difficulties and 7.49% of that on prosocial behavior. While these proportions are modest, they are consistent with the expectation that multiple pathways, including biological, genetic, and other environmental factors, contribute to gender differences in child mental health. The observed indirect effects indicate that parental overreactivity is statistically involved in this association, but they also suggest that the majority of the gender–mental health relationship operates through other mechanisms. However, the cross-sectional design precludes any causal interpretation of the mediation results. The observed indirect effects are statistically consistent with the proposed model, but alternative models (e.g., child mental health influencing parental overreactivity, or unmeasured confounders) cannot be ruled out. Therefore, we present these findings as hypothesis-generating and emphasize the need for longitudinal research to test directionality.

Gender serves as a significant social category that is associated with children’s development and behavior (30). The observed gender differences might be attributed to a combination of biological, psychological, and sociocultural factors. Such patterns likely reflect manifestations of gender-specific social expectations and attitudes that parents transmit through both conscious and unconscious behaviors (31). However, the statistical indirect effect of parental overreactivity is consistent with the possibility that parenting behaviors may play a role in amplifying or mitigating these differences. A possible explanation underlying these associations is gender-differentiated parenting, whereby parents employ distinct strategies toward sons and daughters (32). This behavior might originate from parental gender socialization, a process through which parents convey and reinforce gender-related social expectations to their children. Theoretically, such mechanisms might involve a range of cognitive factors, including intergroup attitudes, gender essentialism, intrinsic parenting motivation free from gender stereotypes, gender identity, and conflict resolution strategies (33). Studies consistently suggest that parents tend to employ harsher, more controlling strategies with sons, including psychologically and physically controlling practices, while using more sensitive and autonomy-supportive strategies with daughters, characterized by warmth, emotional closeness, and encouragement of autonomy (32, 34–38). The observed gender differences in preschool mental health are consistent with prior evidence that girls tend to exhibit greater persistence of mood and anxiety symptoms compared to boys from early childhood onward (3), underscoring the importance of early preventive efforts targeting both genders.

Our findings indicated that boys scored on average 0.04 points higher on the overreactivity scale than girls, while each one-unit increment in parental overreactivity was linked to an 85% elevated risk of total difficulties along with a 34% decrease in higher prosocial behavior. Although statistically significant, the gender difference in overreactivity (β = -0.04 on a 1–7 item-average scale) is very small in absolute magnitude (Cohen’s d ≈ 0.04). The clinical relevance of this difference remains uncertain, and the large sample size (n = 21,366) provided sufficient power to detect even trivial effects. Parental overreactivity—a dysfunctional disciplinary style characterized by coercive, stringent, and controlling behaviors (19, 39)—might be statistically associated with part of the observed gender gap in mental health. Although not considered abusive, such overreactive parenting has been consistently associated with impaired cognitive development and elevated externalizing behaviors in children (40, 41). Moreover, these practices could be part of a cross-sectionally observed negative feedback cycle wherein children’s behavioral difficulties co-occur with parental stress and overreactivity, which may be associated with deteriorating family dynamics. The reverse pathway is equally plausible: children with more difficult temperaments or behavioral problems may elicit harsher parenting responses, consistent with transactional models of development that posit reciprocal influences between parenting and child behavior. Given the cross-sectional design, our data cannot distinguish between these competing directions. Extensive evidence supports the beneficial effects of positive parental behaviors—such as warmth and shared positive affect—on child development (42). Conversely, negative parental affect and harsh disciplinary approaches have been recognized as significant risk factors for adverse developmental outcomes (42, 43). Therefore, our findings suggest that gender-sensitive parenting interventions aimed at reducing overreactive behaviors, particularly in families with boys, could be a hypothesis worth testing in future longitudinal and intervention research.

Subgroup analysis indicated a consistent trend across all populations, although noteworthy differences were not observed in certain subgroups. In terms of total difficulties, the protective association of girl gender was more pronounced among younger children (<5 years old), suggesting that the influence of gender on mental health was more evident in early childhood and might gradually weaken or be overshadowed by other factors as children grew older and underwent further socialization. In families with an annual income below ¥100,000, the mental health advantage of girls remained significant, indicating that gender had continued to play an important independent role in households with relatively limited economic resources. Low-income backgrounds adversely affected the mental health of children, leading to an elevated risk of behavioral and socioemotional problems (44–46). This finding might have stemmed from two key factors. First, high-income backgrounds, such as greater education or income, were linked to less traditional gender role attitudes (47–49), whereas low-income families often adopted more gender-differentiated parenting practices (50). Indeed, as demonstrated in our study, less overreactivity parenting strategies with girls were associated with more favorable mental health outcomes. Second, financial strain heightened parental stress, which might have led to dysfunctional parenting behaviors, such as overreactivity or laxness, ultimately contributing to poorer child mental health outcomes (51, 52). Specifically, economic pressure could destabilize parental emotional well-being, increasing the likelihood of overreactive parenting (53), which was associated with elevated externalizing problems in children, particularly exacerbated under financial stress (54). In terms of prosocial behavior, the positive association with girl gender remained consistent across all subgroups, and the vast majority of subgroups showed statistically significant difference. This suggested that the development of prosocial behavior might have been more universally influenced by gender and was less sensitive to different background factors. None of the interaction tests reached statistical significance, indicating that while these factors might exert some moderating effects, they did not fundamentally alter the basic pattern of the association between child gender and mental health. Therefore, the statistical mediation pattern of parental overreactivity between gender and mental health remained generally consistent across different subgroups, further supporting the potential relevance of parenting practices in the formation of gender differences in early mental health.

### 4.1 Limitations

We also needed to consider some shortcomings of this research. First, the cross-sectional nature of this study limited causality assessment, as the temporal sequence among child gender, parental overreactivity, and mental health remained unclear. The mediation results should be interpreted as hypothesis-generating rather than causal. Future longitudinal research is needed to verify the direction of these links. Second, data were self-reported by one parent per child, potentially introducing reporting bias, social desirability effects, or incomplete representation of parenting dynamics if the non-responding parent exhibited different behaviors. In addition, because all measures were derived from the same parent respondents, shared-method variance may have inflated the observed associations. The absence of teacher- or clinician-reported assessments further limits the objectivity of our findings. Third, the large sample size meant that even very small differences (e.g., the 0.04-point difference in overreactivity scores, measured on a 1–7 item-average scale) reached statistical significance. The clinical meaning of such small effect sizes remains uncertain and should be interpreted cautiously. Future studies with clinically relevant outcome thresholds or replication in independent samples would help clarify whether these statistically significant differences are practically meaningful. Fourth, although multiple covariates were adjusted for, residual confounding from unmeasured factors (e.g., genetic influences or environmental stressors) could not be ruled out. In particular, we did not assess parental mental health, parenting stress (e.g., using a standardized measure such as the Parenting Stress Index), or broader family functioning, all of which may influence both parental overreactivity and child mental health outcomes, and could therefore partially account for the observed associations. While some of our included covariates, such as parental education, household income, employment status, and marital status, may serve as partial proxies for family resources and functioning, they cannot fully substitute for direct measurement of these constructs. Future studies incorporating validated measures of parenting stress and comprehensive assessments of family context would help clarify the independent role of overreactivity after adjusting for these broader factors. Fifth, while the SDQ is a well-validated screening instrument for child mental health, it is a brief measure that may not capture the full spectrum of child psychopathology with the same depth as more comprehensive tools such as the Child Behavior Checklist (CBCL). Future studies incorporating the CBCL or other detailed diagnostic assessments would be valuable to validate and extend our findings, and to provide a more comprehensive characterization of gender-related differences across a broader range of behavioral and emotional domains. Sixth, our findings were obtained from a single geographic region in western China and may not be generalizable to other cultural contexts. Replication in diverse populations is needed to assess the cultural specificity of the observed associations.

## 5 Conclusions

This study provides evidence suggesting that parental overreactivity may statistically explain part of the association between child gender and mental health outcomes in preschool-aged children. Girls scored on average 0.04 points lower than boys on the overreactivity scale, and this gender-differentiated parenting pattern was statistically associated with better mental health profiles among girls, who demonstrated a 12% lower risk of total difficulties and a 21% higher prosocial behavior compared to boys. Mediation analysis indicated that parental overreactivity accounted for 16.22% of the gender-based differences in total difficulties and 7.49% in prosocial behavior. However, due to the cross-sectional design, causal conclusions cannot be drawn. These findings highlight the potential role of gender-differentiated parenting practices in being associated with early mental health development, pointing to a possible need for interventions focused on parental overreactivity, especially in families with sons. Reducing parental overreactivity may thus represent a meaningful approach to lessening early mental health disparities between boys and girls, though longitudinal research is required to test this possibility.

## Data Availability

The data analyzed in this study is subject to the following licenses/restrictions: This data set is still being used for analysis. Please contact the corresponding author regarding access to the full dataset. Requests to access these datasets should be directed to Jie Mi, mijie666@163.com.
