# Supplementary material for: Unveiling gender disparities in mental health among Chinese preschoolers: partial statistical mediation by parental overreactivity
Source: Front Psychiatry. 2026 Aug 19;17:1876731. doi: 10.3389/fpsyt.2026.1876731 (PMC13534034; doi:10.3389/fpsyt.2026.1876731)
Supplement: Supplementary file 1 [file Table1.docx]

Supplementary Table S1. Variance components and intraclass correlation coefficients (ICC) from null models with kindergarten as random intercept

| **Outcome** | **Between‑kindergarten variance** | **Within‑kindergarten variance** | **ICC** | **Model status** |
| --- | --- | --- | --- | --- |
| Total difficulties | 4.64 × 10⁻¹⁴ | 0.1515 | < 0.001 | Singular fit |
| Prosocial behavior | 0.00 | 0.2500 | 0.000 | Singular fit |
| Overreactivity | 0.00 | 1.1690 | 0.000 | Singular fit |

ICC = between‑kindergarten variance / (between‑kindergarten variance + within‑kindergarten variance). For all three outcomes, the between‑kindergarten variance was essentially zero, indicating no meaningful clustering by kindergarten. Therefore, standard regression models (without random effects) are appropriate for the primary analyses.

Supplementary Table S2. Comparison of key results between complete-case analysis and multiple imputation

| **Outcome** | **Complete-case analysis (n = 21,366)** | **Multiple imputation (n = 25,017)** |
| --- | --- | --- |
| Total difficulties (girls vs. boys) |  |  |
| OR (95% CI) | 0.88 (0.82–0.94) | 0.87 (0.82–0.92) |
| Prosocial behavior (girls vs. boys) |  |  |
| OR (95% CI) | 1.21 (1.14–1.28) | 1.20 (1.14–1.26) |
| Parental overreactivity (girls vs. boys) |  |  |
| β (95% CI) | -0.04 (-0.06 to -0.01) | -0.04 (-0.07 to -0.01) |

Note: Multiple imputation was performed using the MICE algorithm with 10 imputations and 20 iterations per imputation. Results were pooled across the 10 imputed datasets using Rubin‘s rules. Odds ratios and confidence intervals were calculated from pooled coefficients. All estimates rounded to two decimal places and highly consistent with complete-case analysis, confirming robustness to missing data.

Supplementary Table S3. Variance inflation factors (VIF) for all predictors in the regression model

| **Variable** | **VIF** |
| --- | --- |
| Child gender | 1.001 |
| Parental overreactivity | 1.011 |
| Parent age | 1.055 |
| Child age | 1.028 |
| Parent gender | 2.307 |
| Education level | 1.303 |
| Occupation | 1.321 |
| Marital status | 1.016 |
| Household income each year | 1.124 |
| Smoking status | 2.252 |
| Alcohol consumption | 1.738 |

Note: All VIF values are far below 2.5, indicating no substantial multicollinearity among predictors.

Supplementary Table S4. Gender differences in continuous SDQ scores (linear regression)

| **Outcome** | **β (95% CI)** | **P-value** |
| --- | --- | --- |
| Total difficulties | -0.434 (-0.566, -0.302) | <0.001 |
| Emotional symptoms | 0.079 (0.029, 0.128) | 0.002 |
| Conduct issues | -0.070 (-0.106, -0.034) | <0.001 |
| Hyperactivity | -0.142 (-0.184, -0.099) | <0.001 |
| Peer challenges | -0.302 (-0.354, -0.250) | <0.001 |
| Prosocial behavior | 0.230 (0.173, 0.287) | <0.001 |

Reference: Boys. Adjusted for parental gender, parental age, child age, education level, employment status, household income, smoking status, alcohol consumption, and marital status.
